# PBP2a substitutions linked to ceftaroline resistance in MRSA isolates from the UK

**DOI:** 10.1093/jac/dkv317

**Published:** 2015-10-12

**Authors:** Ewan M. Harrison, Xiaoliang Ba, Beth Blane, Matthew J. Ellington, Anette Loeffler, Robert L. R. Hill, Mark A. Holmes, Sharon J. Peacock

**Affiliations:** 1 Department of Medicine, University of Cambridge, Cambridge, UK; 2 Department of Veterinary Medicine, University of Cambridge, Cambridge, UK; 3 Clinical Microbiology and Public Health Laboratory, Public Health England, Cambridge, UK; 4 Royal Veterinary College, Hawkshead Campus, University of London, London, UK; 5 Antimicrobial Resistance and Healthcare Associated Infections Reference Unit, Public Health England, London, UK; 6 Wellcome Trust Sanger Institute, Hinxton, UK

Sir,

Ceftaroline is a new cephalosporin antibiotic with activity against MRSA. Binding of this drug to the allosteric domain of PBP2a leads to a conformational change that allows a second molecule of ceftaroline to bind to the active site, blocking its activity.^1^ Recent reports have described MRSA isolates with low-level (>1–8 mg/L) and high-level (>32 mg/L) resistance to ceftaroline.^2–5^ These have been isolated in geographically dispersed locations (Greece, Spain, Switzerland, Thailand and the USA) and have occurred in numerous genetic backgrounds: ST5 [clonal complex (CC) 5], ST228 (CC5), ST239 (CC8), ST247 (CC8) and ST764 (CC5).^2–5^ Low-level resistance has been associated with N146K, E150K and E239K substitutions in the allosteric binding domain of PBP2a.^1–3^ Recent data have shown that the N146K/E150K substitutions mediate resistance by interrupting the allosteric response of ceftaroline binding, preventing a second molecule of ceftaroline blocking the active site.^6^ Higher-level resistance (8 to >32 mg/L) is mediated by E447K and Y446N/E447K substitutions in the ceftaroline-binding pocket of the transpeptidase region of PBP2a.^2,5^ Furthermore, the E447K substitution has been identified in laboratory-generated, ceftaroline-resistant isolates, along with other chromosomal mutations likely to be involved in high-level resistance.^7^

We sought the presence of these mutations in the whole-genome sequence data of 458 MRSA isolates cultured from humans (*n* = 397) or animals (*n* = 61). Isolates from humans were predominantly drawn from the east of England between 1985 and 1987 (*n* = 180) or between 2006 and 2013 (*n* = 191), with the remainder drawn from other regions of England and Scotland between 2010 and 2012.^8,9^ Animal isolates were cultured from dogs (41), cats (3), horses (4) or cattle (13) in the UK.^8^ We identified three isolates (0.66%) that contained substitutions previously reported to mediate ceftaroline resistance (Table 1). Two isolates (ASARM167 and A38) belonging to ST22 (epidemic MRSA-15) had an E239K substitution in PBP2a. ASARM167 was isolated from a patient with bacteraemia at Cambridge University Hospitals NHS Foundation Trust (CUH) in 2008 and A38 was isolated from a canine wound infection in 2006 treated in Wiltshire, south-west England.^8^ Phylogenetic analysis of these two isolates based on core genome SNPs placed them in different clades separated by >120 SNPs (data not shown), indicating that the E239K mutation arose independently in these two isolates. The third isolate (ASARM130) had the N146K substitution in PBP2a, belonged to ST241 (CC8) and was isolated from a patient with bacteraemia at CUH in 2007. This isolate was also noted to have an N204K substitution, which has not been reported previously in isolates with the N146K substitution.^2,3^

**Table 1.** Characteristics of three MRSA isolates with PBP2a substitutions associated previously with ceftaroline resistance

Strain name	Location	Source	Year	ST (CC)	SCC*mec*	PBP2a substitutions	Ceftaroline MIC (mg/L)	Ceftaroline (5 μg) disc diffusion (mm)	Oxacillin (1 μg) disc diffusion (mm)	Cefoxitin (10 μg) disc diffusion (mm)	Penicillin G (1 U) disc diffusion (mm)
ASARM130	CUH, UK	human, bacteraemia	2007	ST241 (CC8)	II	N146K, N204K	0.5	20	6	6	6
ASARM167	CUH, UK	human, bacteraemia	2008	ST22 (CC22)	IVh	E239K	1	20	6	6	6
A38	veterinary practice, Wiltshire, UK	dog, wound infection	2006	ST22 (CC22)	IVh	E239K	1	19	6	6	6

For oxacillin: disc diffusion—susceptible ≥15 mm diameter, resistant ≤14 mm diameter.

For cefoxitin: disc diffusion—susceptible ≥22 mm diameter, resistant ≤21 mm diameter.

For penicillin G: disc diffusion—susceptible ≥25 mm diameter, resistant ≤24 mm diameter.

For ceftaroline: MIC breakpoint is 1 mg/L; disc diffusion—susceptible ≥20 mm diameter, resistant <20 mm diameter.

The effect of these PBP2a substitutions on the ceftaroline resistance phenotype was evaluated for these three isolates using the disc diffusion assay based on EUCAST guidelines^10^ and the Etest (bioMérieux, Lyon, France) according to the manufacturer's instructions. Two isolates were susceptible and one was resistant to ceftaroline by disc diffusion, but all three isolates were susceptible by Etest (Table 1). Although isolates with the N146K substitution have been reported previously to have an MIC of 0.5 mg/L (susceptible), all previously reported isolates with E239K had an MIC of ≥2 mg/L (resistant).^2,4^ The lack of association between a resistant phenotype and the N146K substitution indicates that secondary chromosomal mutations are likely to be involved, as reported previously.^4,7^ The three study isolates were cultured before the clinical introduction of ceftaroline into clinical practice in the USA in 2010 and Europe in 2012, demonstrating that these are natural variants of PBP2a that occur (albeit at low prevalence) even without pressure from ceftaroline use. All previously reported isolates with PBP2a substitutions mediating ceftaroline resistance belonged to CC5 and CC8, which has led to the suggestion that these two lineages might be more prone to such mutations. Our findings suggest that they probably occur in multiple MRSA lineages including the pandemic CC22 lineage, which is important in many parts of the world including Australia and the Middle East and is the dominant MRSA lineage in the UK and much of Europe.

## Funding

This work was supported by UKCRC Translational Infection Research (TIR) Initiative, and the Medical Research Council (Grant Number G1000803) with contributions to the Grant from the Biotechnology and Biological Sciences Research Council, the National Institute for Health Research on behalf of the Department of Health, and the Chief Scientist Office of the Scottish Government Health Directorate and by a Medical Research Council Partnership grant (G1001787/1) held between the Department of Veterinary Medicine, University of Cambridge (M. A. H.), the School of Clinical Medicine, University of Cambridge (S. J. P.), the Moredun Research Institute and the Wellcome Trust Sanger Institute.

## Transparency declarations

None to declare.
